# Boosting Terahertz Photoconductive Antenna Performance with Optimised Plasmonic Nanostructures

**DOI:** 10.1038/s41598-018-25013-7

**Published:** 2018-04-26

**Authors:** Sergey Lepeshov, Andrei Gorodetsky, Alexander Krasnok, Nikita Toropov, Tigran A. Vartanyan, Pavel Belov, Andrea Alú, Edik U. Rafailov

**Affiliations:** 1 ITMO University, St.Petersburg, 197101 Russia; 2 Aston Institute of Photonic Technologies, Aston University, Birmingham, B4 7ET UK; 3 Department of Chemistry, Imperial College London, London, SW7 2AZ UK; 4 Department of Electrical and Computer Engineering, The University of Texas at Austin, Austin, Texas 78712 USA

## Abstract

Advanced nanophotonics penetrates into other areas of science and technology, ranging from applied physics to biology, which results in many fascinating cross-disciplinary applications. It has been recently demonstrated that suitably engineered light-matter interactions at the nanoscale can overcome the limitations of today’s terahertz (THz) photoconductive antennas, making them one step closer to many practical implications. Here, we push forward this concept by comprehensive numerical optimization and experimental investigation of a log-periodic THz photoconductive antenna coupled to a silver nanoantenna array. We shed light on the operation principles of the resulting hybrid THz antenna, providing an approach to boost its performance. By tailoring the size of silver nanoantennas and their arrangement, we obtain an enhancement of optical-to-THz conversion efficiency 2-fold larger compared with previously reported results for similar structures, and the strongest enhancement is around 1 THz, a frequency range barely achievable by other compact THz sources. We also propose a cost-effective fabrication procedure to realize such hybrid THz antennas with optimized plasmonic nanostructures via thermal dewetting process, which does not require any post processing and makes the proposed solution very attractive for applications.

## Introduction

**Figure 1:**
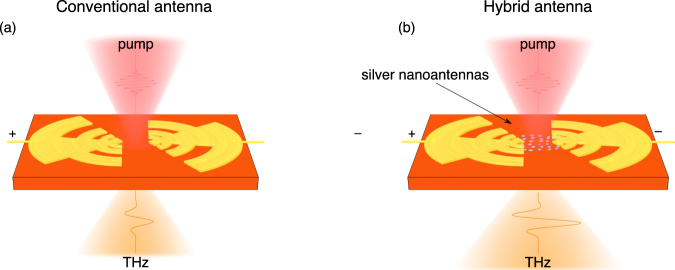
Illustration of the conventional (**a**) and silver nanoantennas-loaded hybrid (**b**) photoconductive THz antenna.

Terahertz (THz) technology is now standing at the lab doorstep to real world applications. The THz spectral band of electromagnetic waves has found a wide range of perspective applications for spectroscopy^1,2^, microscopy^3,4^, terahertz medical sensing^5,6^, security imaging^7,8^, detection of dangerous or illicit substances^8–10^ and ultrafast data transfer^11–13^. However, the widespread use of THz technologies is hampered by the absence of effective, compact and energy efficient THz sources operating at room temperatures. The most common source of *coherent pulsed* THz radiation so far are the so-called THz photoconductive switches, or *photoconductive antennas* (PCAs)^14,15^. A typical realization of such THz antenna in log-periodic design is presented in Fig. 1(a). Here, two (or more) conductive electrodes spaced by a gap are deposited onto a semiconductor surface. The electrodes are biased by an external voltage of several *V*, and the gap between them is pumped by femtosecond (fs) optical pulses. The principles of THz PCAs operation are based on the effect of ultrafast variations of surface photoconductivity of a semiconductor substrate under fs-laser irradiation: after exciting the gap between the electrodes with a fs-laser, the concentration of charge carriers increases sharply for a short period of time, and THz pulse generation occurs.

Since the generated signal is a short single pulse hundreds of fs in duration, its spectrum spreads over several octaves in THz frequency range. The broad bandwidth is ideal for spectroscopic investigations of organic molecules^16^, material science^1,17,18^ as well as wireless THz transmitters and receivers^13^. However, conventional THz PCAs have a rather low conversion efficiency that prevents data transmission over long distances, they do not provide a large signal-to-noise ratio, and require ultrafast laser pump at wavelengths around *λ* = 800 nm, usually provided by expensive and bulky Ti:Sapphire lasers^19^. The low conversion efficiency is mainly related to two predominant factors: the photocarrier screening effect^20,21^, and the low absorption coefficient of the surface layer of the semiconductor substrate. To overcome these limitations, optical nanoantennas^22–24^ have been proposed to be placed in the gap of THz PCA^15,25–28^.

Optical nanoantennas are resonant nanostructures that are capable to transform incident optical waves into a strongly localized near-field^23,29–31^. Nowadays, nanoantennas are commonly used to enhance the absorption coefficient of a semiconductor substrate of PCAs^15,26,32,33^. The nanoantenna-based PCAs have been called *hybrid terahertz-optical PCAs*. The schematic presentation of such antenna is illustrated in Fig. 1(b). It has been shown that this solution provides high fs-laser pump absorption, shorter photocarrier lifetimes, and excellent thermal efficiency^15^. Despite the fact that many nanoantenna designs have been studied for enhancement of the THz generation from PCA, an optimized geometry has not yet been proposed.

Here, we push forward the concept of hybrid terahertz-optical PCAs by comprehensive numerical optimization and experimental investigation of a log-periodic THz photoconductive antenna coupled to a silver (Ag) nanoantenna array arranged in the gap of the THz antenna. We shed light on the operation principles of the resulting hybrid THz antenna providing an approach to significantly boost its performance. By tailoring the size of silver nanoantennas and the distance between them, we obtain an enhancement of optical-to-THz conversion efficiency 2-fold larger in comparison with previously reported results. As a byproduct, we propose a *cost-effective* fabrication procedure allowing to produce such hybrid THz antennas with optimized plasmonic nanostructures via thermal dewetting process.

## Hybrid PCA Design and Fabrication

### Numerical Simulations

**Figure 2:**
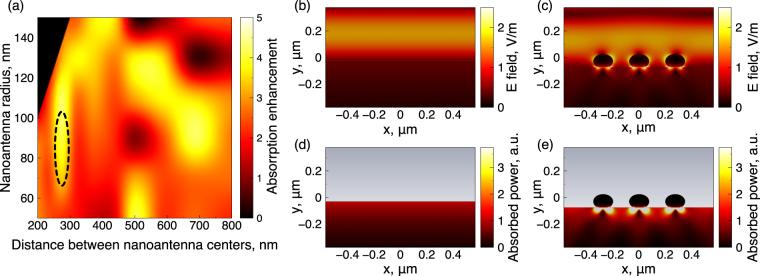
(**a**) Dependence of the absorption enhancement inside the GaAs substrate on the distance between nanoantennas and their radii (D/2). Electric field (**b**,**c**) and density of absorbed power (**d**,**e**) distributions over the pure GaAs substrate and over the substrate containing Ag nanoantennas, correspondingly. The excitation plane wave (with the electric field strength of 1) propagates normally to the surface. The wavelength of excitation is 800 nm.

We start our analysis with numerical calculations of a spheroidal silver (Ag) nanoantenna array arranged over a high-index gallium arsenide (GaAs) semiconductor substrate. Silver was chosen as a material for nanoantennas because it has the smallest dissipative losses among metals in visible and NIR range. Nanoantennas have the form of an oblate spheroid with larger axis D and minor axis d, and these quantities are subject to optimization. It is well-known that such metallic (particularly Ag) nanoparticles provide localized plasmonic resonances in the optical range^34,35^ that manifest themselves in a strong localization of the electric field near the nanoparticle due to conversion of the freely propagating incident waves into the near-field. If the size of the nanoparticles is sufficiently small (in terms of wavelength of the incident wave), the nanoparticle can be treated as an oscillating electric dipole. For the numerical simulations, CST Microwave Studio software package has been used. The calculated absorption enhancement at the wavelength of 800 nm (the wavelength of our fs-laser setup) as a function of the nanoantenna radius (D/2) and distance between nanoantenna centers (a) are presented in Fig. 2(a). Here, the minor radius of the oblate spheroidal nanoparticle (d) has been fixed to 55 nm. Such nanoparticles are typical for our fabrication technique (see bellow). The absorption enhancement has been calculated as the power absorbed in 100-nm GaAs surface film with silver nanoantenna array normalized to the power absorbed in the GaAs film without nanoantennas. The 100-nm GaAs film has been chosen because in this area the external electric field reaches the maximum value^26^. Therefore, the growth of the absorbed power and, thus, carrier density in 100-nm surface layer has the highest impact onto average photocurrent which depends on the external electric field. It leads to enhancement of the photocurrent in THz PCA. Also, in the case of PCA with nanoantennas, the external electric field is additionally increased near the nanoantennas because of extrusion of electric field from the volume of silver nanoparticles.

In Fig. 2(a), it is clearly seen that there are two strong local maxima of the absorption enhancement in the simulation map accompanied by few “hot spots” in the map. The first maximum is in the narrow range of the distances near 280 nm and in the wide range of radii from 70 nm to 115 nm. The second absorption maximum is at the distances of 650–730 nm for the particles with radii ranging between 85 nm and 105 nm. Thus, from these numerical calculations we may expect at least 4-fold enhancement of the THz photoconductive antenna performance with such optimized plasmonic nanostructures. Below we show that such Ag nanostructures may be fabricated via cost-effective method of thermal dewetting, which does not require any post processing. The latter region of high absorption (650–730 nm distance between nanoantenna) is impractical for our case, since the sparse nanoantenna distribution cannot be achieved by laser dewetting that we use for the nanoantenna forming and tuning its size and distribution, hence at nanoantenna fabrication, we aim to the 280 nm spacing and radii of around 90 nm.

The electric field distribution profiles over the air-GaAs interface with and without optimized Ag spheroidal nanoantennas are shown in Fig. 2(b,c). The structure is excited by a normally incident plane electromagnetic wave with the wavelength of 800 nm (the wavelength of the fs-laser used in our setup). The geometrical parameters of nanoparticles correspond to the optimal values obtained from the above mentioned modeling results. The larger axis of the spheroid is D ∼ 170 nm, the minor axis of the spheroid is d ∼ 105 nm. The distance between nanoparticle centers is a = 280 nm. It can be seen that the magnitude of the electric field under the spheroid (Fig. 2(c)) is larger by a factor of 2.5 than the magnitude of the field at the interface between air and GaAs without nanoparticle (Fig. 2(b)). We would like to note that, while nanoparticles are on the surface of a high-index substrate (GaAs), the electric field is mostly concentrated in a thin layer of the substrate surface. The collective interactions excited in the array of nanoparticles cause additional increase of electric field in the gaps between them. These modes, localized and collective, enhance the absorption of fs-laser radiation near nanoantennas and may result in photocurrent density and, thus, THz emission enhancement. The power *P*_abs_, absorbed in a volume $V^{\prime}$ of substrate depends on the electric field distribution inside the substrate and may be estimated as ${P}_{{\rm{abs}}}\sim |E{|}^{2}V^{\prime}$, where *E* is a electric field vector (averaged over the volume $V^{\prime}$) in the medium^15^. Thus, the absorbed power is directly proportional to the value of $|{E}{|}^{2}$ and, therefore, the strong localization of electric field in the semiconductor near the nanoparticles causes an increase of the absorbed power, which results in more effective photoexcitation of the free carriers. Figure 2(d,e) show a comparison of absorbed power densities in the semiconductor with and without nanoparticles. We note that the power absorbed in GaAs with silver nanoantenna is significantly (over 5-times) higher than in pure GaAs near the surface.

### Device Manufacture and Experiment

Next, to prove our theoretical findings, we perform experimental investigations of hybrid PCAs with optimized Ag nanoantennas and compare the obtained results with previous studies^28,36^. The PCAs were fabricated on semi-insulating GaAs substrates containing self assembled InAs quantum-dots (QDs).

Such QD based antennas have been recently demonstrated to generate effectively (though the first batch of these antennas perform not as good as commercially available LT-GaAs PCAs) both pulsed^37,38^ and CW^39^ THz radiation. Since the 800 nm wavelength pump is used, and the carriers are generated in the whole volume of the GaAs matrix, the dots serve only as carrier lifetime shorteners^40^, in a similar way as defects in low temperature grown GaAs.

**Figure 3:**
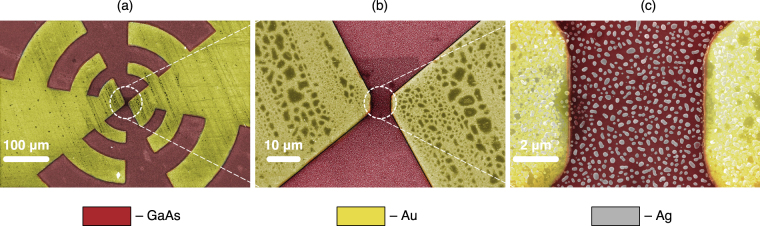
The typical SEM images of the fabricated log-periodic THz PCA (**a**), its central part (**b**), and its gap filled with silver nanoantennas (**c**).

Preparation of the well-ordered periodic array of silver nanoparticles found to be optimal in the numerical simulations is a challenging task. Even in the case of successful realization in the laboratory setting its scalability is doubtful. Because of that we choose the physical vapour deposition as a method to produce the self-organized array of silver nanoparticles that duplicate the structure and the optical properties of the optimized one as well as possible. This process combines the ease of implementation with controllability. The control parameters that enable the resultant film morphology variation are the amount of the deposited material, the deposition rate and the substrate temperature. Additional thermal treatment of the obtained film may be employed for further adjustment of the film morphology. Based on our previous experience^41–44^, we chose the equivalent thickness of the initially deposited silver film to be 20 nm and the deposition rate 2 nm/min. The temperature of the substrate during the deposition process was kept at 250 °C. The subsequent annealing lasted for 1 hour at the same temperature conditions. After cooling the samples were taken out of the vacuum chamber and stored at ambient conditions for weeks without measurable changes of their properties. The scanning electron microscopy (SEM) pictures of the produced hybrid THz antenna are shown in Fig. 3. The sizes of the fabricated nanoparticles approximately correspond to calculated optimal ones and the average distance between nanoparticles is 280 nm. Here, we would like to note that, according to previous studies, neither the enhancing properties of the disordered array differ from the properties of the periodical array insignificantly^45^, nor the size of the plasmonic nanoantennas affects the array absorption resonance value^46^. The dashed oval in Fig. 2(a) shows the location of the resulting nanoantennas on the radius-center distance map. For more details about the structures fabrication see Methods.

**Figure 4:**
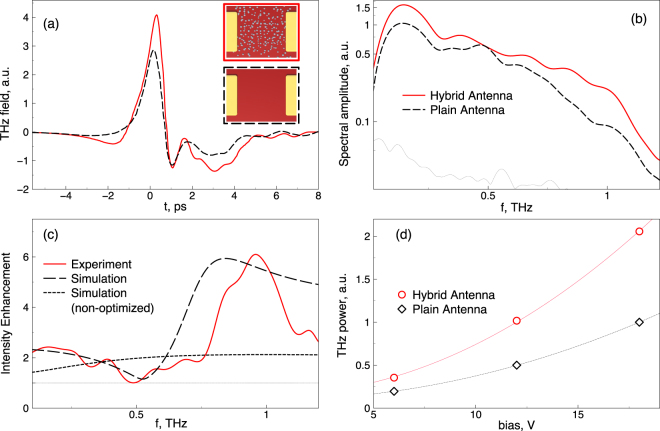
Comparison of the optimized design silver nanoantenna enhanced hybrid PCA performance with the plain PCA. (**a**) Time domain THz field profiles, and (**b**) their corresponding spectral amplitudes. Plain antenna signal is plotted in black, hybrid antenna signal in red, thin dotted curve represents the noise floor in the experiment. (**c**) Spectral intensity enhancement gained due to optimized silver nanoantennas in the gap of the PCA (red line), compared with simulated signal intensity enhancement from hybrid PCA (black dashed line) and simulated enhancement, corresponding to the geometry from the ref.^36^ (black dotted curve). (**d**) Power trends vs bias for optimized hybrid (red) and plain (black) PCAs. Markers correspond to experimentally obtained values, and curves are the $P\propto {V}^{2}$ fits.

For experimental verification of the proposed hybrid antennas, a standard THz time-domain spectroscopic (TDS) system has been used. THz-TDS is pumped with Sprite-XT (M Squared Ltd.) femtosecond Ti:sapphire laser that delivers pulses of 120 fs duration at 80 MHz repetition rate with central wavelength of 800 nm. As a THz detector, a LT-GaAs photoconductive antenna made by Teravil Ltd. was used, and the beam was guided between the transmitter and detector by two off-axis parabolic mirrors. To estimate the effect of silver nanoantennas, signals from standard and nanoantenna-enhanced THz antennas were measured for similar pumping and bias conditions, and revealed practically similar enhancement for pumps between 20 mW–100 mW and biases between 6 V–24 V. The experimental results obtained in ambient environment, at 50 mW of pump power, focussed pump spot size of 30 *μm* in diameter, thus giving the energy density of $\sim 75\,\mu J\cdot c{m}^{-2}$ and 18 V bias voltage, are summarized in Fig. 4.

## Results and Discussion

Figure 4(a) shows the time domain THz signals generated by the log-periodic PCA with and without Ag spheroidal nanoantennas for 12 V bias voltage. The corresponding signal amplitude spectra, obtained using the Fourier transform, are shown in Fig. 4(b). Noise floor in Fig. 4(b) is derived by applying the the time window that selects the leading part of the timescan, before the THz pulse, prior to taking a Fourier transform. It can be seen that the antenna demonstrates an uneven enhancement across the spectrum. The spectrum of THz signal spectral intensity enhancement by optical nanoantennas is shown in Fig. 4(c). The enhancement reaches its maximum around 1 THz, and the enhancement value corresponds to the ∼5-fold theoretically derived value of the power absorption enhancement. Lower effect at other frequencies and negligible enhancement at 0.5 THz can be associated with the change in the THz antenna impedance induced by highly conductive silver in the gap shown in Fig. 4(c). To explain the uneven enhancement, we calculate the frequency dependent signal emitted from the PCA into the far field for nanoantenna enhanced and plain PCAs, as described in Eq. (2) of ref.^47^, and then normalize one by another, correspondingly:

1 $${ {\mathcal E} }_{THz}(\omega )={\eta }^{2}\frac{{G}_{{\rm{plain}}}^{2}+{(\omega {C}_{{\rm{plain}}}+{B}_{{\rm{ant}}})}^{2}}{{G}_{{\rm{optim}}}^{2}+{(\omega {C}_{{\rm{optim}}}+{B}_{{\rm{ant}}})}^{2}}\mathrm{.}$$

Here, ${ {\mathcal E} }_{THz}(\omega )$ is the frequency dependent enhancement of THz generation, *η* is the normalized PCA substrate surface susceptibility to the optical pump, i.e. pump absorption enhancement, calculated earlier, *G*_plain_ and *G*_optim_ are the real parts of plain and optimized PCA conductivity, *C*_plain_ and *C*_optim_ are the corresponding capacities, and *B*_ant_ is the complex antenna conductivity. All impedance parameters were retrieved from frequency domain PCA simulation in COMSOL Multiphysics. It is worth noting, that spectral shape of the enhancement is predominantly defined by the imaginary conductance values, while real part is responsible for the magnitude. The simulated THz enhancement curve for our sample is shown in black dashed curve, while the curve for the antenna from the ref.^36^ are shown in Fig. 4(c). Apparently, uneven enhancement in our sample occurs due to the change of gap impedance. The enhancement previously shown for similar type, but not optimized hybrid PCA in ref.^36^ lacks these spectral features, and our calculations confirm this behaviour. The overall power of generated THz signal with (red curve) and without (black dashed curve) nanoantennas under the 50 mW pump are plotted as a function of the PCA bias in Fig. 4(d). The results demonstrate significant amplification of the emitted THz signal spectral intensity with increasing of the bias voltage. The results for THz PCAs with nanoantennas demonstrate over 5-fold increase in comparison with the case of plain PCA without nanoantennas. This is 4 times higher than the results that have been previously reported for the spectral intensity^36^. Overall generated THz power enhancement is greater than 100% (over than 2-fold), which is twice the enhancement in the above mentioned publication.

## Conclusions

In conclusion, we have demonstrated, both theoretically and experimentally, an unprecedented enhancement of the photoconductive antenna operation efficiency by optimized spheroidal Ag plasmonic nanoantennas. The resulting hybrid PCA demonstrates over 5-fold increase in the generated THz signal around 1 THz, and over 2-fold increase in overall generated THz power, which coincides with our theoretical estimations and overcomes previously demostrated results for the approach. Moreover, we have proposed the cost-effective fabrication procedure to realize such hybrid THz antennas with optimized plasmonic nanostructures via thermal dewetting process, which does not require any post processing and makes the proposed solution very attractive for applications. We believe that our results may be useful for many relevant applications, requiring compact and effective room-temperature THz sources, including spectroscopy, biological sensing, security imaging, and ultrafast data transmission.

## Methods

### QD substrate growth

The PCA GaAs substrate containing self-assembled InAs QDs was grown by molecular beam epitaxy (MBE) in the Stranski-Krastanov regime on a GaAs substrate. The QDs were set in 40 layers, resulting in overall active region thickness of ∼1 $\mu$m. Each QD layer was grown by deposition of 2.3 monolayer (ML) InAs at 500 °C, capped by 4 nm ${{\rm{In}}}_{0.15}{{\rm{Ga}}}_{0.85}{\rm{As}}$ then by 6 nm GaAs, after which the temperature was raised to 580 °C before growth of the subsequent 30 nm GaAs spacer layer, in order to desorb segregated In. Also, prior to growth of each QD layer, the GaAs surface was annealed under an As_2_ flux for 5 minutes to flatten the growth surface. AFM analysis gives an estimated QD density of around $3\cdot {10}^{10}$ cm^−2^ per layer.

### PCA Electrodes deposition

Metallic antenna electrodes were deposited over a semiconductor substrate using a standard UV photolithography and further wet etching of the surface Ni/Au (40 nm/200 nm, respectively) features. Post-process annealing to increase Ohmic contact between the antenna metal and GaAs surfaces was applied. The log-periodic shape of electrodes has been selected for the realization of the THz antenna because such design provides a broadband radiative spectrum, thus, the only reason that kept the generated spectrum relatively narrow if compared to commercial PCAs, were longer carrier lifetimes in QD PCA wafers^40^. The fabricated THz antennas have a smallest gap of 8 *μ*m, and overall diameter of 1.8 mm.

### Silver nanoantennas growth and tailoring

Physical vapor deposition of silver (99.99% purity) onto the surface of the fabricated nanoantennas was performed in PVD-75 (Kurt J. Lesker) vacuum chamber at residual pressure of ${10}^{-7}\,mbar$. Usually plasmonic nanoparticles are prepared in two steps. The first step is the physical vapor deposition of the carefully chosen amount of silver on the substrate kept at the room temperature. The obtained metal film has a semi-continuous labyrinth structure with very broad and shifted in the IR plasmon band^48^. The second step consists in thermal annealing that activates surface diffusion and finally leads to dewetting and formation of a 2D array of well separated nanoparticles. The annealing temperature and the duration of annealing as well as the amount of the deposited material, the deposition rate and the substrate temperature during the deposition are the control parameters that enable the resultant film morphology variation. The position of the plasmon band dependence on the amount of deposited material was studied in the case of quartz substrate in ref.^44^ and in the case of GaAs substrate in^41^. Based on the vast experience in silver nanoparticles fabrication and investigation of their plasmonic properties^41–44,48^ we chose the particular set of control parameters that led to the successful approximation of the optimal film morphology suggested by the numerical simulations. The equivalent thickness of the silver film as measured *in situ* by the quartz crystal microbalance was equivalent to 20 nm, while the deposition rate was set to 2 nm/min. We found also that rise of the substrate temperature during the deposition process has similar effect on the final morphology of the film as the prolonged annealing. Because of that the substrate temperature was kept at 250 °C during the deposition process. The subsequent annealing lasted for 1 hour at the same temperature conditions. After cooling the samples were taken out of the vacuum chamber and stored at ambient conditions for weeks without measurable changes of their properties. According to scanning electron microscopy (SEM) investigations, run several weeks after the samples were taken out of the vacuum chamber, the average lateral diameter of the islands was about 150 nm. Surface density was estimated to be 10^9^ cm^−2^. Such conditions of deposition and thermal annealing provide both coupling of quantum dots with plasmonic excitations and did not shunt the substrate with the THz antenna on its surface.

### THz-TDS setup

THz-TDS setup consisted of plain/silver enhanced QD PCA under study as a THz transmitter, and a LT-GaAs PCA (Teravil Ltd.) as a THz detector. The beam was guided between the transmitter and detector by two off-axis parabolic mirrors. THz-TS setup was pumped with Ti:sapphire femtosecond laser (Sprite-XT by M Squared Ltd.) capable to deliver 120 fs pulses at 80 MHz repetition rate with central wavelength of 800 nm. The pump laser beam was focussed into spot with a size of $30\,\mu m$ in diameter, thus giving the energy density of $\sim 75\,\mu J\cdot c{m}^{-2}$ for 50 mW pump power.

### Numerical Simulation

The power absorption of optical pump was simulated using a commercial Software (CST Microwave Studio). In the model, the nanoantenna radii were varied in the range 50–150 nm, and the distance between them in the range 200–800 nm, where possible. A plane wave is falling onto the plain/enhanced semiconductor surface at normal incidence. The resulting spatial distribution of the electromagnetic field and absorbed power density was computed by using a finite elements approach (FEM) in the entire simulation volume. Gap impedance of plain and nanoantenna enhanced PCAs was calculated using a frequency domain solver in commercial software (COMSOL Multiphysics).
